# Current Peptide and Protein Candidates Challenging HIV Therapy beyond the Vaccine Era

**DOI:** 10.3390/v9100281

**Published:** 2017-09-29

**Authors:** Koollawat Chupradit, Sutpirat Moonmuang, Sawitree Nangola, Kuntida Kitidee, Umpa Yasamut, Marylène Mougel, Chatchai Tayapiwatana

**Affiliations:** 1 Division of Clinical Immunology, Department of Medical Technology, Faculty of Associated Medical Sciences, Chiang Mai University, Chiang Mai 50200, Thailand; kool_krub@msn.com (K.C.); sutpirat_mo@hotmail.com (S.M.); umpa_119@hotmail.com (U.Y.); 2 Center of Biomolecular Therapy and Diagnostic, Faculty of Associated Medical Sciences, Chiang Mai University, Chiang Mai 50200, Thailand; goy_ahsup2012@hotmail.com (S.N.); kitidee_010@hotmail.com (K.K.); 3 Institute of Research in Infectious Diseases, CNRS UMR9004, University of Montpellier, 34293 Montpellier, France; 4 Division of Clinical Immunology and Transfusion Sciences, School of Allied Health Sciences, University of Phayao, Phayao 56000, Thailand; 5 Center for Research and Innovation, Faculty of Medical Technology, Mahidol University, Nakhon Pathom 73170, Thailand

**Keywords:** HIV, HIV gene therapy, HIV vaccine, assembly inhibitor, entry inhibitor, fusion inhibitor, integration inhibitor

## Abstract

Human immunodeficiency virus (HIV) is a causative agent of acquired immune deficiency syndrome (AIDS). Highly active antiretroviral therapy (HAART) can slow down the replication of HIV-1, leading to an improvement in the survival of HIV-1-infected patients. However, drug toxicities and poor drug administration has led to the emergence of a drug-resistant strain. HIV-1 immunotherapy has been continuously developed, but antibody therapy and HIV vaccines take time to improve its efficiency and have limitations. HIV-1-specific chimeric antigen receptor (CAR)-based immunotherapy founded on neutralizing antibodies is now being developed. In HIV-1 therapy, anti-HIV chimeric antigen receptors showed promising data in the suppression of HIV-1 replication; however, autologous transfusion is still a problem. This has led to the development of effective peptides and proteins for an alternative HIV-1 treatment. In this paper, we provide a comprehensive review of potent anti-HIV-1 peptides and proteins that reveal promising therapeutic activities. The inhibitory mechanisms of each therapeutic molecule in the different stages of the HIV-1 life cycle will be discussed herein.

## 1. Introduction

Currently, it is estimated that around 37 million people live with human immunodeficiency virus (HIV), the virus that causes acquired immunodeficiency syndrome (AIDS) [1]. Generally, HIV can be divided into two types, HIV-1 and HIV-2, and are distinguished by genetic differences and AIDS pathophysiology. The main clinical difference between these two types is that AIDS progression is much slower in HIV-2 infection when compared with HIV-1 infection. Both HIV-1 and HIV-2 are the results of zoonotic transfers of viruses infecting non-human primates in Africa [2]. The major target cell for HIV-1 infection is the CD4^+^ T lymphocyte [3]. However, other cells are also susceptible to the virus, especially macrophage, and dendritic cells [4]. There are other potential tissues, including the kidney [5,6], liver [7], lung [8], breast [9], brain [10,11], and hematopoietic stem cells [12,13], that HIV-1 can also infect. Furthermore, the virus can persist silently in the body as an HIV reservoir.

Anti-retroviral drugs are the standard treatment for viral load suppression and for the reduction in mortality of the target cells leading to a longer lifespan for HIV-1-infected patients. Importantly, anti-retroviral drugs can also reduce the risk of HIV-1 transmission. More than 25 antiretroviral drugs approved by the Food and Drug Administration (FDA) are being used. These drugs target different steps of the HIV-1 life cycle. A highly active antiretroviral treatment (HAART) is the standard drug regimen that combines several potent antiviral agents for HIV-1-infected patients. Even though people can easily access antiretroviral drugs, whose delivery is fully facilitated by the government, antiretroviral therapy can fail to suppress the plasma viral load. The emergence of HIV-1 drug resistance (HIVDR) is mainly caused by poor medication adherence resulting in viral rebound in peripheral blood. Skipping or not taking the antiretroviral dose correctly can increase the occurrence of HIV drug resistance because, when the amount of drugs in the body becomes low, the virus can reproduce freely and accumulate more mutations. Today, two platforms of antiviral administration have been applied to reduce the risk of HIV-1 infection among HIV-negative people: pre-exposure prophylaxis (PrEP) and post-exposure prophylaxis (PEP). The first, PrEP, is for HIV-1 seronegative people who are at risk of contracting the virus, for example female or male sex workers [14]. Another aspect of using short-term antiretroviral drugs, or PEP, is to take the drugs immediately after contact with an HIV-positive sample, had an accident during a medical operation, or had unsafe sex with a partner [15]. However, the effectiveness in the reduction of new HIV-1-infected cases using these two strategies does not reach 100%. Some data have shown a significant reduction in HIV-1 transmission, but it is not 100% effective in preventing HIV-1 infection in cases of sexual exposure [16].

Furthermore, HIV-1 latency in cell populations, e.g., memory T lymphocytes and macrophages is one of the hindrances of using antiretroviral drugs to eradicate integrated proviruses from resting cells [17]. There are several possible mechanisms involved in the persistence of HIV-1 reservoirs: (i) the long half-life of HIV-1-infected resting T cells [18]; (ii) the insufficiency of antiretroviral drugs in lymphoid tissue compartments where the viruses are replicating [19]; and (iii) the possibility for the virus to spread through cell-to-cell transmission [20]. These are the main reasons for being unable to eradicate HIV reservoirs from the infected patients. Therefore, alternative treatments to antiretroviral drugs (ARV) have been proposed as HIV-1 gene therapy, not only to overcome the bottleneck of using ARV, but also to eradicate the infected cells or HIV reservoirs. In this review, we will describe the development of the advancements of peptide and protein-based molecules for HIV-1 treatment.

## 2. HIV-1 Vaccine Development

During the HIV pandemic, the development of HIV vaccines started when highly broad neutralizing antibodies (bNAbs) were considered as a passive vaccine to counteract HIV-1 infection. Despite the neutralizing antibodies being discovered right after the disease breakthrough, specific antibodies produced in HIV-infected patients were unable to neutralize all mutant HIV strains and resulted in a high level of escape strains regarding a high mutation rate [21].

Neutralizing antibodies were designed to target many sites on the HIV-1 envelope glycoprotein (Env), which is important to bind CD4 molecules to the target cell surface. The strategies relied on the immune system, which generates bNAbs after infection, and involved in virus-host co-evolution and host tolerance mechanisms [22,23]. There were five major target sites for the bNAbs: (i) the V2 site within the V1V2 domain [24], (ii) the N-linked site at N332 located downstream of the V3 loop [25], (iii) the CD4 binding site of envelope glycoprotein 120 (gp120) [26], (iv) the epitopes in the gp120-gp41 interface [27], and (v) the membrane proximal external region (MPER) of gp41 [28]. The first generation of bNAbs were a group of antibodies, such as b12, 2G12, 2F5, Z13, and 4E10, that targeted the envelope spike consisting of their epitopes spanning along gp120 and gp41. They have been shown to have functions in in vitro neutralization and to protect against simian/human immunodeficiency virus (SHIV) in macaques in vivo [29]. However, there were limitations in using bNAbs given that a high concentration of these neutralizing antibodies was required to achieve a high efficacy against a wide range of HIV-1 infection.

The PGT121-class bNAbs—targeting the N332-glycan domain—was boosted by an engineered stabilized trimeric envelop of HIV as an immunogen [30]. Unfortunately, the HIV-1-resistant strain could escape from the N332-directed NAb by extension into the V1 loop of up to 21 amino acids [31]. This resistance limited its potential use for HIV-1-infected patients. The second generation of bNAbs, VRC01 and PG9/PG16, showed strong neutralizing activities and high efficacies with a ten-fold lower number of antibodies required when compared to the first generation of bNAbs [32]. Recently, 3BNC117, targeting the CD4 binding site on the viral envelope, showed promising HIV-1 protection in animal models and decreased the viral load in HIV-1-infected patients [33,34]. The Fc-engineered VRC01 antibody was designed to promote the phagocytic activity of these phagocytes within the tissues [35].

The neutralizing antibodies mentioned above serve as potential models for a HIV-1 vaccine, so many active vaccines have been developed in the past several decades. The HIV-1 vaccine trials, VAX003 and VAX004, did not prevent HIV-1 infection in high-risk volunteers [36,37]. RV144 was a vaccine clinical trial that combined the canarypox vector (ALVAC-HIV) and a boost of the AIDSVAX p120 vaccine. ALVAC is an attenuated non-replicating viral vector, while AIDSVAX B/E consists of recombinant proteins derived from HIV-1 CRF01_AE and B subtypes. Both have been tested for their safety [38,39,40], and lower rates of HIV infection have been observed with a decrease of 31% at 42 months when compared to the control group [41]. This vaccine was thought to demonstrate these effects through antibody-dependent cellular cytotoxicity (ADCC), but has since been shown a low efficacy within a short period of time, which has made it less promising. However, the reasons for the failures of this vaccine were most likely from the variability of the viral envelope proteins that made this vaccine ineffective [42]. The use of adenovirus vectors, STEP (HVTN502) and Phambili (HVTN503), as delivery methods failed to protect, and seemed related to an increased risk of HIV-1 infection in some of the population [43,44].

Furthermore, there are multiple obstacles for inducing bNAbs as antiviral agents; for example, the designed immunogens display imprecise epitopes leading to inefficient bNAbs production and the glycosylation of the HIV envelope heavily covers the conserved sites of the envelope that are the binding sites of most bNAbs [45,46]. The establishment of HIV-1 vaccines appears to be very difficult, so there has not been a fully effective vaccine available until now. A summary of HIV-1 vaccine developments is presented in Table 1.

## 3. Alternative HIV Gene Therapies

Based on the limitations in using HAART and the failure of vaccines, new approaches of HIV-1 alternative treatments have been developed and are described here in more detail.

### 3.1. Adoptive T Cell Transfer and Immunotherapy

Adoptive T-cell therapy (ACT) has been developed to overcome many diseases including cancer. There are also many strategies that have been investigated to reduce the viral load in HIV-infected individuals. T lymphocytes can be modified by gene-transfer to stably express therapeutic genes to enhance immune function. Autologous immune cells, usually the T cells of patients, are modified by altering the specificity of the T-cell receptor (TCR), or introducing chimeric antigen receptors (CARs) to T cells. There are several methods that can introduce therapeutic genes into human T lymphocytes including nonviral-based delivery (DNA transfection) or viral-based delivery (retrovirus, lentivirus, adenovirus, or adeno-associated virus (AAV)). Some studies have been undertaken where T cells were modified to combat HIV-1, one of which being the use of CARs.

Using CAR T cells for HIV therapy is an alternative approach to fight HIV-1. The first model was a CD4-based CAR that designed receptor binding to gp120 on the surface of infected cells and suppressed HIV-1 replication. It consisted of CD4 extracellular and transmembrane domain fused with the CD3ζ intracellular signaling domain [47]. Preclinical studies of *CD4ζ* gene-modified CD4^+^ and CD8^+^ T cells showed it was safe, but had no significant reduction in viral level [48]. This may have been due to several factors including the rapid loss of CAR expression, the CD4 molecule being used by the virus to infect cytotoxic T cells (CTLs) [49], or a low expression of gp120 on the infected cells.

Several neutralizing antibodies recognizing various sites of the HIV envelope glycoprotein have been isolated and characterized. In 2016, Ayub Ali et al. generated a single chain construct CAR based on seven bNAbs against HIV-1 that showed the ability to recognize HIV-1-infected cells, kill, and suppress viral replication [50]. Moreover, in 2017, Malika Hale et al. combined CAR technology including a gene-editing tool to generate human T cells expressing CARs and knock out C-C chemokine receptor type 5 (CCR5) molecules on the cell surfaces. Several single chain variable regions (scFvs) derived from bNAbs were used to develop potent anti-HIV CARs with different epitopes of the HIV-1 envelope glycoprotein. To disrupt CCR5 molecule expression simultaneously, they introduced a CAR gene into a CCR5 locus in primary T cells. The results showed an efficient reduction of viral replication when compared to a single HIV CAR expression. The benefit of this strategy was not only to target HIV-1-infected cells, but also prevent the effector cells from HIV-1 infection [51]. However, one limitation is that C-X-C chemokine receptor type 4 (CXCR4)-tropic HIV-1 may still have the ability to infect target cells.

Despite CAR T-cell immunotherapy appearing to be the most impressive method to treat HIV-1-infected patients, there are still some limitations. The CAR T cells can be targeted by the humoral and cellular immune responses of patients, which can cause anaphylaxis reactions in humans [52]. The expansion of autologous T cells results in the promotion of infected cell numbers and is prone to serious treatment problems. Moreover, a previous study has shown that ex vivo expanded autologous HIV-specific cytotoxic T cells (CTLs) cannot completely reduce the virus, which was probably due to rapid cell death and the lack of specificity [53].

### 3.2. Peptides and Proteins for HIV Therapy

**Figure 1:**
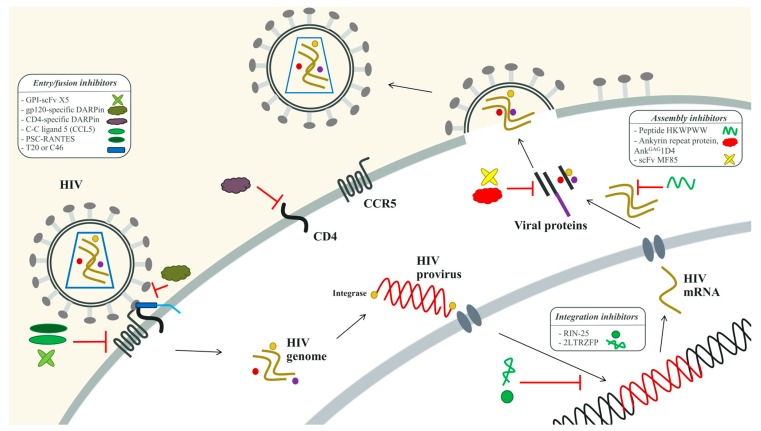
Summary of functional sites of anti-HIV peptide and protein inhibitors at different stages of the HIV-1 life cycle. For further details please refer to the text.

Several viral molecules involved in all steps of the HIV-1 life cycle can be possible targets. There are two anti-HIV-1 proteins, classified as immunoglobulin- and non-immunoglobulin-based structures: scFv and ankyrin repeat proteins (DARPins), respectively. This review focused on the application of current peptides and proteins as intracellular antiviral molecules for HIV-1 therapy as seen in Figure 1.

#### 3.2.1. The HIV-1 Entry Inhibitors

The entry process begins with the adhesion of viral gp120 glycoprotein to the host-cell-specific molecule—in this case, the CD4 molecule, which contributes to the fusion of cell and viral membranes. Once the gp120 binds to the CD4 molecule, conformational change will occur where the gp120 subunit (called the hyper variable loop) is revealed to bind to the co-receptor, CCR5 or CXCR4. After the V3 and co-receptor bind, the gp41 conformational changes subsequently result in membrane fusions and the internalization of the viral capsid into the host cell.

For immunoglobulin-based agents, scFv X5 (derived from anti-HIV-1 Env X5) can confer resistance in human primary CD4 T cells to HIV-1 by expressing the cell surface via a glycosylphosphatidylinositol (GPI) anchor. Primary CD4 T cells expressing GPI-anchored scFv X5 have been shown to be resistant to CCR5, CXCR4, and dual-tropic HIV-1 strains ex vivo. In a hu-PBL mouse study, GPI-scFv X5-transduced CD4 T cells reduced viral loads when compared to the control group [54]. The designed ankyrin repeat proteins (DARPins)—non-immunoglobulin-based agents—share the same binding specificity with their target as with the antibody, and show higher properties in terms of stability, solubility, and well-expression inside cells [55]. DARPins are different to antibodies because of their smaller size and structure. They consist of 33 amino acids in each repeat unit that can specifically bind to target proteins using their surface of α-helices and β-haipins, which form into a groove-like binding surface [56].

A gp120-specific DARPin is a viral entry inhibitor as it specifically recognizes the V3 loop region [57]. A CD4-specific DARPin specifically interacts with CD4 molecules, resulting in the inhibition of HIV-1 entry [58]. These ankyrin repeat proteins have shown promising properties in recognizing their targets with high affinity and specificity. However, the binding of gp120-specific DARPin to gp120 drives HIV-1 mutations in the HIV envelope and CD4-specific DARPin can cause losses of CD4 T-cell function, thus leading to unwanted side effects. A previous study has shown that CD4-specific DARPins can recognize rhesus CD4 and inhibit SIV infection in vitro, but cell-free DARPins were rapidly cleared from circulation, which was one drawback in using this protein [59].

On the other hand, the Regulated upon Activation, Normal T cell Expressed and Secreted (RANTES) and C-C ligand 5 (CCL5) are natural anti-HIV-1 inhibitors that target the CCR5 molecule [60]. PSC-RANTES, an analog of chemokine RANTES, showed potent protection against HIV-1 vaginal transmission in monkey models [61]. Interestingly, R4.0, a peptide derived from human CCL5/RANTES to obtain higher anti-HIV-1 activity, showed an IC_50_ value that nearly matched that of Enfuvirtide (T20) [62]. Moreover, the combination of the CCL5-derived peptide R4.0 with various classes of HIV-1 inhibitors such as Maraviroc (MVC) demonstrated concomitant bindings with CCR5 and increased the effect of HIV-1 inhibition in acute HIV-1 infection assays [63].

Even though blocking CCR5 molecules can prevent HIV-1 R5 tropism in the target cells, it may have negative effects in controlling other chronic infections, for example, central nervous system (CNS) infectious diseases [64]. Furthermore, the fusion inhibitors, T20 and C46, are synthetic C-terminal peptides derived from the gp41 C-terminal heptad repeat (CHR). While T20 contains only 36 amino acids, C46 consists of 46 amino acids; however, both overlap the sequence of the C-terminal heptad repeat of HIV-1 gp41. These peptide inhibitors mimic the activity of heptad repeat 2 (HR2) by competitively interacting with heptad repeat 1 (HR1), leading to the interference of conformational change of gp41. As a result, the HIV-1 viral membrane cannot fuse with the target cell membrane [65]. Accordingly, soluble T20 is an antiviral peptide approved by the FDA for treatment of chronic HIV-1 infection. C46 has been improved to be more effective than T20 since T20 can develop HIV-1-resistant strains. A combination of membrane C46 with other molecules such as shRNA-CCR5 has shown promising results in HIV-1 protection in many cell types in vitro and in vivo [66,67]. This combination, developed by Calimmune Inc. (Pasadena, CA, USA), is still in phase I/II trials; however, some reports have demonstrated that the mutation of gp120 can contribute to C46-resistant strains named HIV_Bal_C46 [68].

#### 3.2.2. The HIV-1 Integration Inhibitors

The integrase enzyme (IN) plays a major role in inserting reverse transcribed viral cDNA into the host genome. The integration process can be divided into two steps: first, 3′-end processing, the cleavage of a dinucleotide from 3′ end of each strand of the viral DNA [69]; and second, DNA strand transfer where viral DNA integrates its DNA into the target host chromosome [60]. Step 2 can be inhibited by strand transfer inhibitors raltegravir, elvitegravir, and dolutegravir; however, HIV drug-resistant strains by amino acid substitution can be found [70].

Indolicidin, an antimicrobial tridecapeptide amind, was found to have anti-HIV-1 activity [71]. Moreover, RIN-25, an indolicidin derivative, has been found to have an IN-inhibitory effect and is also more potent than the original indolicidin [72]. This indolicidin peptide has antiviral activities against envelope viruses, but is toxic to host cells. 2LTRZFP, a non-immunoglobulin-based structure, was designed as a zinc finger protein to specifically target the end terminal of HIV-1 LTR to block HIV-1 genome integration [73,74]. The major benefit of this molecule is that it might function with all HIV-resistant strains as mutation at 2LTR does not occur. Since the integrase enzyme recognizes the conserved region of 2LTR, if some mutations occur at this area, it will not be able to complete the integration process, thus resulting in no viral replication. Moreover, these proteins do not interact or downregulate host proteins that may affect the function of normal cells. Therefore, this molecule is an interesting candidate for developing a potent strategy for HIV-1 gene therapy in the future.

#### 3.2.3. The HIV-1 Assembly Inhibitors

HIV assembly, budding, and release are essential steps in late-stage viral replication and involve a concomitant HIV Gag and GagPol processing. The nucleocapsid (NC) domain at the C-terminal of the Gag precursor is a small basic domain with nucleic acid binding activity. The nucleocapsid contains two zinc finger motifs that coordinate zinc ions and are flanked by short flexible linker peptides. Furthermore, NC is involved in many steps such as reverse transcription [75], RNA encapsidation [76], and viral assembly [77,78]. Thus, NC can be one of the major targets in HIV therapy [79].

The peptide competitor, HKWPWW, selected by using phage-displayed peptide libraries, was shown to specifically bind to the packaging signal (named ψ) of genomic RNA, impairing the RNA incorporation into nascent particles, resulting in the reduction of HIV-1 infectivity [80,81,82]. Another approach is to prevent the interaction of NC with its nucleic acid partners using a cyclic peptide designed to imitate the mature nucleocapsid protein 7 (NCp7). Additionally, an antiviral effect was observed in HIV-1 replication in infected human T-lymphoblastoid-4 cells [83].

Furthermore, the multimerization of Gag proteins at the plasma membrane plays a key role in the virus assembly process. Gag–Gag interaction initiates in the cytoplasm and once at the plasma membrane, Gag continues to multimerize through a poorly understood mechanism [84]. The scFv, MF85, which binds to HIV-1 p24 antigen, was constructed using phage display technology. It showed similar properties as monoclonal antibodies with a high binding affinity at 2 nM [85]. However, one limitation of using scFv given that the cytoplasm is a reducing condition is that it may not be suitable for protein folding and the biological function of this molecule. This makes non-immunoglobulin-based molecules more advantageous than immunoglobulin-based molecules. An ankyrin repeat protein named Ank^GAG^1D4, a non-immunoglobulin-based molecule, has been designed to target the capsid (CA) domain of the HIV-1 Gag. It binds to the N-terminal domain of the CA (NTDCA), resulting in the inhibition of the HIV-1 assembly process. In addition, the study showed that Ank^GAG^1D4 could strongly inhibit SIVmac and SHIV replication in the SupT1 cell line [74,86].

However, when HIV has already integrated its DNA proviral into the host chromosome (in cases of HIV-1 heavy infection), the scFv MF85, peptide competitor, or Ank^GAG^1D4 probably have limited effects. Indeed, they probably would not completely inhibit or block the virus assembly, resulting in the release of low amounts of new virions enabling de novo infection. A summary of peptides and proteins tested for HIV-1 therapy is provided in Table 2.

## 4. Conclusions

The HIV/AIDS pandemic is still a major concern as the number of infected individuals has significantly increased in recent years. Although HAART can suppress the viral load in peripheral blood, there are some subpopulations of cells that are infected and remain silent, making the antiretroviral drugs unable to eliminate the virus. The development of an HIV-1 vaccine is still difficult, and there has been no effective vaccine available until now. Additionally, current findings show that the use of rhesus monkey cytomegalovirus (RhCMV)-based simian immunodeficiency virus (SIV) vaccine can directly prime major histocompatibility complex class-E (MHC-E)-restricted CD8^+^ T-cell responses. The unusual pathway of this antigen presentation can possibly extend the success of HIV vaccine in the future [87]. Adoptive T-cell transfer and immunotherapy have limitations on cell transfusion and take a longer period of time to modify and expand cells. Therapeutic peptides and proteins are becoming alternative strategies for HIV-1 treatment as there are several advantages of using macromolecules as inhibitors, for example, they have high specificity and affinity towards their molecular targets and HIV-1 mutants have difficulty evading these molecules.

The future development of therapeutic peptides and proteins for HIV-1 therapy seems promising. Some of them have already demonstrated interesting characteristics in in vitro and in vivo animal experiments. Some antiviral proteins are currently being further investigated in animal models prior to entering clinical trials. Enfuvirtide (T20) has been used as an HIV-1 fusion inhibitor and has been combined with other antiretroviral drugs to increase the viral inhibitory effect in HIV-1-infected individuals. However, it has been reported that T20 can contribute to HIV-1 mutant strains. C46 is a fusion inhibitor that has been improved to overcome HIV resistance toward T20, but this molecule has been also been reported regarding mutant HIV-1. A combination of C46 with other molecules such as 2LTRZFP or Ank^GAG^1D4 might ameliorate the protection in HIV-1 C46 mutant strains and can be a possible future trend for HIV-1 gene therapy.

Additionally, peptides and proteins are not the only interesting candidates for alternative HIV-1 therapy. Biofluid exosomes are vesicles released from many cell types into extracellular space. Some of them have been proven to have antiviral activities. Exosomes purified from breast milk can inhibit HIV-1 infection in dendritic cells (DC) by binding to dendritic cell specific intercellular adhesion molecule 3 (ICAM-3) grabbing non-integrin (DC-SIGN) resulting in blocking viral transfer. Semen-derived exosomes can inhibit HIV reverse transcriptase (RT) activity leading to the reduction in viral RNA when compared to viruses generated from the lack of semen-derived exosomes [88]. In the HIV maturation process, the integrase enzyme also binds to viral RNA during viral maturation to protect viral RNA and IN from degradation. The use of allosteric integrase inhibitors (ALLINIs) could reduce the stability of viral RNA, thus blocking the HIV-1 reverse transcription process [89].

Based on our knowledge of the bacterial defense system, the clustered, regularly interspaced, short palindromic repeats (CRISPR)/CRISPR-associated 9 (Cas9) nuclease has also been used as tools for gene editing. It has been reported that using RNA-guided CRISPR/Cas9 targeting HIV LTRs can eliminate integrated HIV-1 DNA from HIV-1-infected human CD4^+^ T cells, therefore leading to the inhibition of HIV infection [90]. Moreover, it has been demonstrated that CRISPR-based genetic screening can identify five host dependency factors (HDFs), which are essential for HIV infection. Inactivation of these genes may confer resistance to viral infection, and this approach can be applied to other pandemic and epidemic viruses [91].

However, there are both advantages and disadvantages in using these HIV-1 inhibition molecules, but they need improvements for future use in HIV-1-infected patients. The current peptide and protein developments mentioned in this review put forward the hope that by using these molecules, HIV-1-infected patients can be possibly cured in the near future.

## Figures and Tables

**Table 1:** Human immunodeficiency virus type 1 (HIV-1) vaccine developments.

Vaccines	Types	Results	Immunogens	References
VAX003	Passive vaccine	No vaccine efficacy	gp120 of HIV-1 subtypes B and E (strains MN and A244)	[36]
VAX004	Passive vaccine	No vaccine efficacy	gp120 of HIV-1 subtypes B (strains MN and GNE8)	[37]
STEP (HVTN502)	Active vaccine: T-cell activation	No vaccine efficacy, increased HIV infection rates	Subtype B, MRKAd5-*gag/pol/nef*	[43]
Phambili (HVTN503)	Active vaccine: T-cell activation	No vaccine efficacy	Subtype B, MRKAd5-*gag/pol/nef*	[44]
RV114	Passive and active vaccine	Estimated efficacy 31% at 42 months	Prime: subtype B and A/E ALVAC-HIV-gag-pr-gp41-gp120 Boost: subtypes B and E AIDSVAX B/E (gp120 subunit proteins)	[40,41]

**Table 2:** Peptide and protein candidates for HIV-1 therapy.

Peptides or Proteins	Types	Targets	Efficacy	Clinical Trials	References
	**Immunoglobulin-Based Molecules**				
GPI-scFv X5	Entry inhibitor	CCR5, CXCR4 co-receptor	Protects CD4^+^ T cells from R5, X4, and dual-tropic HIV-1; can be immunogenic and generate HIV-1 mutants in long-term infections	Preclinical trial	[54]
scFv MF85	Assembly inhibitor	HIV-1 p24	High binding activity towards p24 antigen; not suitable for protein folding in cytoplasm	Ongoing research	[85]
	**Non-Immunoglobulin-Based Molecules**				
gp120-specific DARPin	Entry inhibitor	HIV-1 gp120	Target gp120 with high affinity and specificity; would drive HIV-1 mutation in HIV-1 Envelope	Ongoing research	[57]
CD4-specific DARPin	Entry inhibitor	CD4 molecule	Targets CD4 with high affinity to inhibit HIV entry; rapidly cleared from the circulation	Ongoing research	[58,59]
C-C ligand 5 (CCL5)	Entry inhibitor	CCR5 co-receptor	Binds to CCR5 molecule to inhibit HIV entry	Ongoing research	[60]
PSC-RANTES	Entry inhibitor	CCR5 co-receptor	Inhibits HIV-1 vaginal transmission in monkey model	Preclinical trial	[61]
T20	Fusion inhibitor	HIV-1 gp41, NHR	Antiviral peptide approved by FDA for inhibiting HIV-1 entry; has to inject twice daily and can develop HIV-1-resistant strains	Phase II	[65]
C46	Fusion inhibitor	HIV-1 gp41, NHR	More effective than T20; can develop HIV-1-resistant strains	Phase I/II	[66,67,68]
RIN-25	Integration inhibitor	HIV-1 IN	Exhibits IN-inhibitory activity	Ongoing research	[72]
2LTRZFP	Integration inhibitor	HIV-1 LTR	Targets HIV-1 2LTR to block HIV-1 integration	Ongoing research	[73,74]
Peptide competitor, HKWPWW	Assembly inhibitor	HIV-1 ψ-RNA	Inhibits HIV-1 assembly by binding to packaging signal of genomic RNA	Ongoing research	[80,81,82]
Ankyrin repeat protein, Ank^GAG^1D4	Assembly inhibitor	HIV-1 p24	Binds to N-terminal of HIV-1 capsid, limited effects in HIV-1 heavy infection	Ongoing research	[74,86]
